# The ANTI-Vea-UGR Platform: A Free Online Resource to Measure Attentional Networks (Alertness, Orienting, and Executive Control) Functioning and Executive/Arousal Vigilance

**DOI:** 10.3390/jintelligence11090181

**Published:** 2023-09-08

**Authors:** Tao Coll-Martín, Rafael Román-Caballero, María del Rocío Martínez-Caballero, Paulina del Carmen Martín-Sánchez, Laura Trujillo, Luis Cásedas, M. Concepción Castellanos, Klara Hemmerich, Greta Manini, María Julieta Aguirre, Fabiano Botta, Andrea Marotta, Elisa Martín-Arévalo, Fernando G. Luna, Juan Lupiáñez

**Affiliations:** 1 Mind, Brain and Behavior Research Center (CIMCYC), University of Granada, 18071 Granada, Spain; tcoll@ugr.es (T.C.-M.); rrarroca@ugr.es (R.R.-C.); fabianobotta@ugr.es (F.B.); marotta@ugr.es (A.M.); emartina@ugr.es (E.M.-A.); 2 Department of Research Methods in Behavioral Sciences, University of Granada, 18071 Granada, Spain; 3 Department of Experimental Psychology, University of Granada, 18071 Granada, Spain; 4 Instituto de Investigaciones Psicológicas (IIPsi, CONICET-UNC), Facultad de Psicología, Universidad Nacional de Córdoba, Córdoba 5010, Argentina

**Keywords:** vigilance, attention, online assessment

## Abstract

The Attentional Networks Test for Interactions and Vigilance—executive and arousal components (ANTI-Vea) is a computerized task of 32 min duration in the standard format. The task simultaneously assesses the main effects and interactions of the three attentional networks (i.e., phasic alertness, orienting, and executive control) and two dissociated components of vigilance with reasonable reliability (executive and arousal vigilance). We present this free and publicly accessible resource (ANTI-Vea-UGR; https://anti-vea.ugr.es/) developed to easily run, collect, and analyze data with the ANTI-Vea (or its subtasks measuring some attentional and/or vigilance components embedded in the ANTI-Vea). Available in six different languages, the platform allows for the adaptation of stimulus timing and procedure to facilitate data collection from different populations (e.g., clinical patients, children). Collected data can be freely downloaded and easily analyzed with the provided scripts and tools, including a Shiny app. We discuss previous evidence supporting that attention and vigilance components can be assessed in typical lab conditions as well as online and outside the laboratory. We hope this tutorial will help researchers interested in measuring attention and vigilance with a tool useful to collect data from large sample sizes and easy to use in applied contexts.

## 1. Attentional Networks and the ANTI-Vea

### 1.1. What Is the ANTI-Vea Task?

One of the most widely used approaches for the understanding of human attention is the integrative model developed by Michel I. Posner (Petersen and Posner 2012; Posner and Petersen 1990; Posner and Dehaene 1994; Posner 1994). According to this framework, attention can be considered as a system exerting three different attentional functions: alertness (or selection in time), orienting (or selection in perception), and executive control (or selection at response levels), all playing an important overall role in behavioral coordination. These attentional functions are modulated by three neural networks (Fernandez-Duque and Posner 2001): a network involving frontal and parietal regions of the right hemisphere modulated by noradrenergic release for alertness, a posterior network (frontal eye fields, parietal cortex, and other subcortical structures) modulated by cholinergic innervations for orienting (Corbetta 1998), and two anterior circuits (fronto-parietal and cingulo-opercular systems) modulated by dopaminergic activity for executive control.

Following the original Attention Network Test (ANT; Fan et al. 2002), specific adapted versions of the ANT have been developed aiming to improve the assessment of the three attentional functions (for a review, see Almeida de Souza et al. 2021). For example, the ANT for Interactions (ANTI; Callejas et al. 2004) task allows measuring not only the way the networks work but also how they interact with each other. Furthermore, whereas executive control is measured similarly with the ANT and the ANTI, the ANTI (and following developments like the ANTI-V and ANTI-Vea) measures phasic alertness and orienting differently. An auditory warning signal, instead of the visual cue used in the ANT, is used to measure phasic alertness with the ANTI. On the other hand, whereas a central vs. a 100% valid peripheral cue is used to measure orienting with the ANT, a peripheral non predictive cue is used to measure orienting with the ANTI version.

A subsequent version, the ANTI-Vigilance (ANTI-V; Roca et al. 2011) was developed with the aim of adding a direct measure of maintenance of attention over time-on-task, that is, vigilance. Additional adaptations of these tasks have been developed, incorporating new components and adjusting them to specific populations, such as children (e.g., Child ANT, Rueda et al. 2004; ANTI-Birds, Casagrande et al. 2022) or patients with visual impairments (Auditory ANT, Johnston et al. 2019; Roberts et al. 2006). For a more detailed review of the origins and different evolutions of the task, see Almeida de Souza et al. (2021).

Since the development of the original ANT by Fan et al. (2002), our team has contributed substantially to this enterprise. We are currently working on the dissemination of the latest version, developed during the last five years: the ANT for Interactions and Vigilance–executive and arousal components (ANTI-Vea; Luna et al. 2018). The ANTI-Vea is suitable to assess the performance of the attentional networks and their interactions, and it provides two independent measures of vigilance, in line with other well-known tasks like the Sustained Attention for Response Task (SART; Robertson et al. 1997) and the Mackworth Clock Test (MCT; Mackworth 1948) for executive vigilance (EV) or the Psychomotor Vigilance Test (PVT; Dinges and Powell 1985) for arousal vigilance (AV). The platform we have built for the use of the ANTI-Vea and the embedded subtasks is user-friendly, and the data, despite being complex and providing multiple measures, can be easily analyzed with the provided guide and resources. The present tutorial aims to present a detailed description of the ANTI-Vea-UGR platform, introducing a theoretical and methodological description of the ANTI-Vea and then a step-by-step guidance on the use of the online ANTI-Vea task and its different available resources for data analyses.

### 1.2. ANTI-Vea Relevance: Dissociation between Executive and Arousal Vigilance

When measuring vigilance, the behavioral pattern usually observed depicts a decrease in performance across time-on-task (Al-Shargie et al. 2019; Doran et al. 2001; Mackworth 1950; Tiwari et al. 2009). Theoretical and empirical research has proposed a dissociation between two components of this ability: (a) EV, understood as the capacity to monitor and detect critical signals that rarely occur over a long period of time, and (b) AV, which refers to the ability to maintain a fast response to any stimulus in the environment (see Luna et al. 2018). Thus, while the EV decrement has been observed as a gradual loss in the hit rate in the MCT and the SART (See et al. 1995; Thomson et al. 2016), the AV decrement has been instead reported as a progressive increase in the average and variability of reaction time (RT) in the PVT (Basner et al. 2011; Lamond et al. 2008; Loh et al. 2004). These behavioral patterns describe the so-called vigilance decrement.

The relevance of the ANTI-Vea relies on allowing a simultaneous (yet independent) assessment of the EV and AV components in a single experimental session. In this task, the EV component is assessed with a signal-detection task similar to the MCT (Mackworth 1948), in which participants have to discriminate the vertical displacement of the central arrow. In turn, the AV component involves a reaction time task akin to the PVT (Dinges and Powell 1985), in which participants must stop a countdown as quickly as possible. While the EV decrement is observed as a decrease in both hits and false alarms (FAs), leading to an increase in response bias rather than a loss of sensitivity^1^, in line with Thomson et al. (2016), the AV decrement is characterized as an increase in mean and variability of RT.

### 1.3. A Summary of the ANTI-Vea Design

The standard ANTI-Vea combines three classic attentional and vigilance paradigms in three different types of trials, which allows measuring the functioning of the three attentional networks and their interactions (ANTI trials) while simultaneously testing the decrement in executive and arousal vigilance across time-on-task (EV and AV trials, respectively). The three types of trials are randomly presented within each block of trials.

The largest proportion of trials (ANTI trials, 60%) are similar to the ones used in the ANTI, based on a classic flanker task (Eriksen and Eriksen 1974), but also incorporating attentional orienting (as in a spatial cueing paradigm; Posner 1980) and alertness (an auditory tone as a warning signal) manipulations. The remaining trials are evenly distributed between the two vigilance paradigms. As described above, for the assessment of the EV component, participants have to discriminate the vertical displacement of the central arrow (EV trials, 20%), while in the case of the AV component, participants must stop a countdown as quickly as possible (AV trials, 20%).

**Figure 1:**
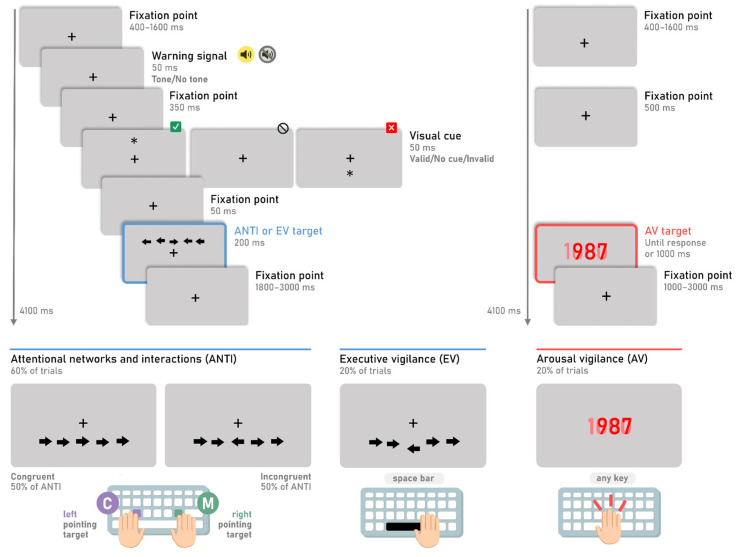
ANTI-Vea trials and correct responses. The top left panel shows the temporal sequence of the ANTI and EV trials. Target and flankers may appear above or below the fixation cross and point to the left or the right side with equal probability. The warning signal appears in half of these trials. The visual cue (i.e., asterisk) has an equal chance of appearing in the same location as the target (trial marked with a green check mark), in the opposite location (marked with a red cross), or not appearing (marked with a barred symbol). The bottom left and middle panels show the correct response based on whether the target is vertically aligned with the flankers (ANTI trials) or displaced (EV trials). The right panels show the temporal sequence and correct response of the AV trials. The duration of each task interval appears next to its corresponding box. Note that although each trial lasted 4100 ms, the sequence of events appeared at a variable interval within each trial.

The general procedure is represented in Figure 1. Further details on the trials procedure can be found in Luna et al. (2018) as well as in the “Method” section in the website. While participants keep their eyes on a black fixation point (“+”) that remains centered on the screen the whole time, a horizontal string of five black arrows appears for 200 ms either above or below the fixation point. Participants are instructed to indicate the direction of the central arrow (i.e., the target) by pressing the corresponding correct key, while ignoring the direction of the surrounding arrows (i.e., the distractors). RT and percentage of errors of congruent and incongruent trials allow the assessment of executive control (also referred to as “cognitive control” or “executive attention”). The target can be anticipated by an auditory warning signal, suitable to test phasic alertness, and by a non-predictive visual cue, suitable for measuring orienting.

The EV trials (i.e., the trials of the signal-detection subtask of the ANTI-Vea) follow the same procedure and stimuli presentation as the ANTI trials. However, the target appears vertically displaced (either upwards or downwards) from its central position in relation to the alignment with the flankers (for a detailed description on the displacement of target and distractors, see the “Method” section of the website and Luna et al. 2018). To complete the EV subtask correctly, participants are instructed to remain vigilant at all times to detect the large vertical displacement of the target and to press the space bar regardless of the target’s direction. Note that the target displacement is considered as the infrequent critical signal of the signal-detection task in the ANTI-Vea. Thus, if participants correctly detect the target’s displacement in EV trials, the response is categorized as a hit. If this displacement is not detected (i.e., the space bar is not pressed), the response is categorized as a miss. Instead, if participants press the space bar in the ANTI trials, the response is categorized as a FA (i.e., an incorrect detection of the infrequent critical signal).

Lastly, for the AV trials (i.e., the trials that mimic the PVT), no warning signal, visual cue, or flankers are presented. Instead, as can be observed in the right panel of Figure 1, the fixation point remains on the screen until a red millisecond countdown appears at the center of the screen, starting at 1000 and descending to 0 or until a response is executed. Participants are instructed to remain vigilant at all times and to stop the millisecond countdown every time it appears on the screen as fast as possible by pressing any key on the keyboard. The AV is thus evaluated with the mean and variability of the RT to the countdown.

The standard ANTI-Vea includes a four-block practice phase, in which instructions and visual feedback are provided so that participants can gradually familiarize themselves with each type of trial. A detailed description of the practice blocks can be found in Luna et al. (2018).

Once participants complete the practice phase, six consecutive experimental blocks are run, without pause and visual feedback. The total time of the experimental blocks is 32 min 48 s for the standard format of six blocks (5 min 28 s per block; 21 min 52 s in the sometimes used four-blocks version). Further details on the design of the ANTI-Vea can be found in Luna et al. (2018) as well as in the “Method” section of the website. For a better understanding of what this experimental phase looks like, a video is available on the website (direct link at https://videopress.com/v/0hmK7b0Q).

In some versions of the task or when some additional parameters are used (see Section 3.1. Summary of Features and Options), other types of trials are added to the task (also randomly within each block of trials). Thus, in the ANTI-Vea-D version of the task, eight additional trials are added per block in which a salient image of a cartoon character is added to measure distraction by irrelevant but salient information. Similarly, it is possible to add a variable number of thought probes (TP) to each block to measure mind wandering across time on task. In these trials, participants have to answer the following question: “Where was your attention just before the appearance of this question?” Participants respond by moving the cursor on a continuous scale ranging from “completely on-task” (extreme left, coded as −1) to “completely off-task” (extreme right, coded as 1). It is possible to select the option of 4, 8, or 12 TPs per block. The presentation of the TP trials is pseudo-randomized, so that there are at least five trials of the ANTI-Vea task between TPs.

### 1.4. ANTI-Vea Indexes

The complex structure and multiple manipulations present in the ANTI-Vea allow for obtaining a wide variety of attentional functioning indexes. The core indexes of the ANTI-Vea comprise 8 attentional network scores (ANTI) and 10 vigilance scores (EV and AV). These core indexes are described in Table 1.

ANTI scores include both mean RT and error rate for the overall ANTI trials, as well as the phasic alertness, orienting, and congruency (i.e., executive control) effects. For RT in ANTI trials, incorrect trials and RTs below 200 ms or above 1500 ms are usually filtered out, which complies with Luna et al. (2021a). Vigilance scores include both overall performance indexes and their decrement slope across task blocks. The EV measures are the percentage of hits and FAs, whereas the AV scores are the mean RT, the standard deviation (SD) of RT, and the percentage of lapses. Note that for FAs, only a set of ANTI trials (i.e., ANTI trials with more than 2 px of random noise from the target to at least one of its two adjacent flankers; referred to as the FA difficult column in the trial dataset) are considered. This allows for the emergence of a decreasing trend of FAs across blocks due to the avoidance of a floor effect (Luna et al. 2021a). The analytical method for computing FAs in a subset of ANTI trials, aiming to avoid a floor effect in FA rate, can be reviewed in detail in Luna et al. (2021b).

## 2. Reliability of the ANTI-Vea Measures

Table 2 summarizes the findings about the internal consistency scores found for the ANTI-Vea core indexes. In terms of internal consistency, a recent study conducted by Luna et al. (2021a) provides consistent evidence that the ANTI-Vea task (administered either in the lab or as an online session) is roughly as reliable as the ANT (MacLeod et al. 2010) and the ANTI-V (Roca et al. 2018) for the measurement of the classic attentional networks. As for EV and AV, while most of the overall scores (i.e., the average performance on the entire task) showed acceptable internal consistency (i.e., split-half correlations corrected by the Spearman–Brown prophecy > 0.75) in both lab and online settings (Luna et al. 2021a), the vigilance decrement scores (i.e., the linear slopes of each vigilance outcome across the six blocks of the task) are substantially less reliable (Cásedas et al. 2022; Coll-Martín et al. 2021; Luna et al. 2022). Even so, as observed by Coll-Martín et al. (2023), these measures of decrement are reliable enough to achieve satisfactory statistical power with a sample size of *N* = 292, a sample size more feasible to achieve thanks to our platform.

Previous studies have also shown that the ANTI-Vea is suitable to be used in repeated sessions, thus supporting the stability of the task’ scores. In Sanchis et al. (2020), participants completed the ANTI-Vea in the lab in six repeated sessions. Although some EV and AV scores were modulated by experimental manipulations (i.e., caffeine intake and exercise intensity), most of the task’ scores were not modulated in the experimental sessions. To specifically assess the stability of the online ANTI-Vea, we conducted a pre-registered study in which 20 participants completed the online task across ten repeated sessions (https://osf.io/vh2g9/; unpublished data). Preliminary analyses showed that main effects of phasic alertness and executive control were not modulated across sessions. Most importantly, the drop in hits for EV and the increase in mean RT for AV were also not modulated across sessions. Interestingly, as observed in Ishigami and Klein (2010) for the ANT and ANTI tasks, split-half reliability scores of the online ANTI-Vea increased as a function of the number of sessions.

## 3. Online Version: The ANTI-Vea-UGR Platform

Having explained what the ANTI-Vea is and what it measures, as well as having demonstrated the reliability of its measures, we now explain the different characteristics of the platform our team has developed, in which contexts the ANTI-Vea can be applied, and, above all, how to collect, analyze, and interpret the data. Note that the current versions of the task on the platform are only available to be administered via computer.

The ANTI-Vea-UGR platform (https://anti-vea.ugr.es) is a research resource offered freely to researchers interested in investigating attention. Different programming languages have been used in its design: JavaScript ES5, HTML5, CSS3, and Angular JS. This allows researchers to freely collect data in the laboratory or online with the available task versions. Additionally, they can download the scripts of these attentional tasks in different programming languages to adapt or modify the existing versions. Although not typically the case, it should be noted that researchers can choose to administer the online version of the task in a lab setting or to send participants the offline (i.e., downloaded) version of the task for them to run it outside the lab.

It is possible to run the complete ANT-Vea task with the ANTI, EV, and AV trials, or to run the tasks with all stimuli but with participants only having to respond to some specific trial types (ANTI, EV, and AV as single tasks, as well as EV-AV as a dual task). Thus, it is possible to run a version in which participants have to respond just to ANTI trials, thus providing only the main measures of the three attentional networks. Similarly, it is possible to run versions of the task in which, although all trial types are presented, participants have to respond only to either EV trials (SART) or to AV trials (PVT), therefore only providing measures of EV and AV, respectively. In addition, the same task versions are provided, but with only the corresponding trials being presented (i.e., presenting only one specific type of trial; ANTI-Only, PVT-Only, SART-Only-Go, SART-Only-NoGo). Two versions are provided for the SART as a function of whether participants are to respond to all trials except for the displaced arrow trials (SART-Only-NoGo) or to only the displaced arrow trials (SART-Only-Go). Note that the SART and PVT tasks as provided in the ANTI-Vea-UGR platform are adapted versions from the original ones running with the specific parameters of the ANTI-Vea task.

Furthermore, it is possible to run an ANTI-Vea version with eight additional trials per block in which a salient image of a cartoon character is added to measure irrelevant distraction (ANTI-Vea-D). The addition of the salient image does not seem to affect the measurement of the other attentional indexes of the ANTI-Vea (Coll-Martín et al. 2021, 2023).

Finally, the tasks can be run with the standard parameters (presented in Table 3) or with some variations of these parameters (e.g., without practice, with more or fewer blocks of trials, with varying degrees of difficulty, and with longer or shorter stimulus duration).

Although experimental conditions (i.e., environmental noise, luminosity, the device on which the task is run) cannot be controlled as much as in the laboratory (Luna et al. 2021a), this platform is effectively addressing the growing need for online method administration and self-reporting for the collection of large data samples (Germine et al. 2012). This alternative has great advantages, being easy to use in applied contexts. In terms of time and cost-efficiency, the online version of the task is far less expensive (e.g., no need for a laboratory infrastructure and no need for a person to explain the task individually to each participant) and allows data to be collected from participants from anywhere in the world. It should be emphasized that the online version of the ANTI-Vea has been proven to be as reliable as the standard one in assessing the main effects, the interaction, and the independence of the classic attentional components, along with the overall performance and decrement in EV and AV (Cásedas et al. 2022; Coll-Martín et al. 2021; Luna et al. 2021a).

In the studies conducted in our lab/research group, the procedure was conducted under the same conditions for all participants: individually in an experimental room, using headphones, using the same device, and under the same conditions of luminosity, distance from the screen, etc. When participants perform the task online, they can do it from home or from any suitable place of their choice, as long as there is a good internet connection. In order to reduce any distractions along the process, the online version of the ANTI-Vea includes additional instructions at the beginning. Participants are warned that the task will be displayed in full screen and that it is important to complete it without interruptions or pauses, and that any other entertainment devices (TV or radio) have to be turned off. The instructions suggest setting the device volume to 75% and to turn off cell phones or set them to silent mode. The experimenter may also monitor the session by video call and instruct the participants to ensure the correct understanding and performance of the task. Group sessions can also be conducted using the ANTI-Vea platform, as the online server supports more than one session at a time.

### 3.1. Summary of Features and Options

The standard ANTI-Vea and its versions on the platform can be run in six different languages, namely, Spanish, English, German, French, Italian, and Polish. This is a remarkable feature, as it allows the user to study the attentional functioning within and across different countries and cultures. We are open to incorporating additional languages to expand the free access of attention assessment into diverse populations.

**Figure 2:**
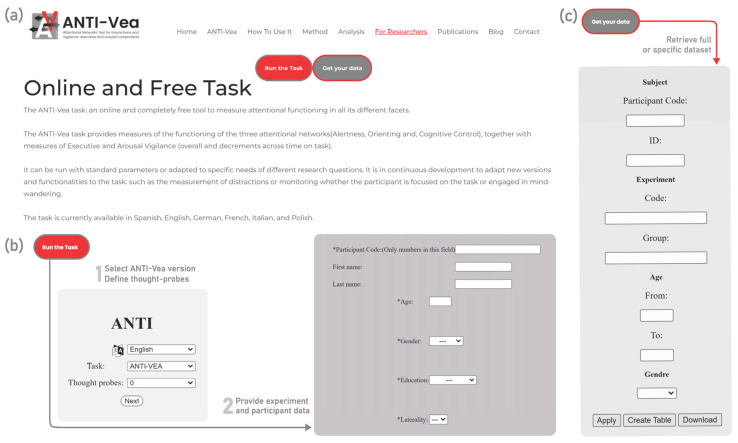
(**a**) Home page of the ANTI-Vea-UGR platform. https://anti-vea.ugr.es/. (**b**) Website for data collection for the online version of the ANTI-Vea and other versions and subtasks. Accessed via the “Run the task” button on the ANTI-Vea-UGR platform. (**c**) Website for downloading experimental data from the ANTI-Vea Task. Accessed via the “Get your data” button on the ANTI-Vea-UGR platform website.

On the website, you can find specific sections with different features of the task. The *Home* section (Figure 2a) presents a brief description of the task, as well as statistics on website visits, years in use, number of participants, published papers related to the task, available languages of the task, and countries using the tool worldwide. The webpage also offers more detailed information about the task and the different ANTI-Vea versions. There is also a *How to use it* section and other useful menu items such as *ANTI-Vea Method*, *Analysis*, *For Researchers*, *Publications*, *Blog*, and *Contact*.

On the “How to use it” section of the website, you can find all the know-how knowledge that is necessary to collect data with the task, how to analyze it, and how to interpret it. To collect data with the online ANTI-Vea or any of its versions—regardless of whether you use the online website either inside or outside the lab—one needs to click on the red button “Run the task”, which will bring one to the online website for data collection (Figure 2b). There, the individual (or the participants) will have to select the appropriate parameters of the task and introduce the corresponding code for each experiment and participant. Some settings can be modified before starting the task (see Table 3). In the Settings window, one can also uncheck the “Do practice blocks” to run the task without practice (only the instructions are presented to remind the participants of the response keys). This is useful when conducting a study in which one manipulates any within-participant variable (e.g., exercise level, time of day, caffeine intake, etc.). In this case, one can ask participants to first perform the whole task in a first familiarization session, and then do it as many times as necessary, according to their within-participant experimental conditions, but without the practice blocks (for an example of this procedure, see Sanchis et al. 2020).

### 3.2. Data Collection, Protection, and Download

In the *For Researchers* section, we provide a system for each user to create a customized and unique link for each participant to access the task. Table 3 shows all the adjustable setting parameters that can be customized to make the task fit your experimental procedure. Note that this is an advanced option for the data collection process. This option is useful when the experimenter wants to avoid giving participants control over the selection of task settings. For example, the experimenter may want to ensure that all participants correctly write their code (unique for each participant) and the name of the experiment (the same for all participants in a given study), as well as perform the practice trials plus just four experimental blocks.

It must be noticed that, in the current version of the ANTI-Vea-UGR platform, several individuals can execute the task with the same values in the Participant code, Experiment, and Group fields. Although the likelihood that the same values in these fields would be repeated in different studies might be small, researchers may want to take into consideration some recommendations to facilitate the identification of the downloaded data as the one of their study. To start with, researchers should be aware that, even if two or more participants share the same codes in the Participant Code, Experiment, and Group fields, the data collected will not be overwritten. This is given that, for each task execution, a unique Subject ID (i.e., an identifier automatically generated by the system for each participant) is assigned for data storage. When possible, researchers can ask participants to send the Subject ID of the task execution after completing the task (in fact, this is usually done for controlling the payment or course credits assignment that participants receive). Moreover, although the Experiment and Group fields are currently not mandatory to execute the task, we strongly recommend researchers to assign original codes on these fields in their studies, in order to avoid potential duplicates of these codes between different studies. Lastly, researchers should also be aware that the date and time of data collection is stored and downloaded for each task execution. Thus, researchers can check if the task was completed within the period that the study was in course or when it was supposed to be completed by that participant. To further facilitate researchers the identification of the data of their studies as well as to avoid potential duplicates in the combination of the Participant Code, Experiment, and Group fields, the team of the ANTI-Vea-UGR platform will continue working in new modifications on this web-tool.

As for the protection of the data, it should be reminded that the provision of identifiable information (i.e., name and surname) is optional and in any case will not be available for download. Furthermore, our platform’s server is managed by the University of Granada, a public institution that adheres to data protection policies and maintains strict ethical guidelines in line with standard academic practices. Researchers can request the removal of their participant data or study information from our database at any time, and participants have the right to request deletion or access to their data. To ensure a participant’s understanding of how their data will be collected and treated, their rights during and after participation, the study’s objectives, and any other relevant aspects, researchers must provide them with detailed information in this regard and an informed consent that ensures their understanding and agreement must be signed prior to participation.

Once the data have been collected, if one is to click on the gray “Get your data” button (located right next to the red “Run the task” button mentioned in the previous step), they will be able to download your raw data file in CSV format. Here, they need to enter the specific details they have used during the collection (typically, the Experiment Code) and click on the “Download” button (Figure 2c). The “Analysis” section of the website presents a detailed description of the raw data files that are downloaded after data collection. A detailed description of each column and their values from a raw data file can be found in the document provided in the website (https://anti-vea.ugr.es/download/codebook_data_trial.xlsx).

In the raw data file, each row contains the information corresponding to each single trial of the task. Some columns show the participant’s details entered at the beginning or selected in the setting window and some extra details automatically provided by the system, such as the Subject ID^2^ the Session Number (automatically generated by the system based on the Subject ID) and the Session Date (yyyy-mm-dd hh:mm:ss).

## 4. Analyzing Data: Scripts and Tools for Analysis of Data

In the *Analysis* section, one will find instructions and tools to analyze their data. In this section, open access scripts can be downloaded for analyzing data collected with the ANTI-Vea. Further scripts will be uploaded in the future with new data analysis on the task.

Starting from the downloaded raw dataset from the “Get your data” section, the analysis procedure typically begins with a pre-processing phase. Note, however, that what follows is a description of the standard procedure, but researchers may choose to follow alternative analytic strategies depending on their specific research aims. Here, practice trials are removed and participants with incomplete experimental blocks, minimization of the task (i.e., unintentional exits from full-screen task display mode leading to incorrectly registered trials), and poor performance are identified. Note that the raw data allow exclusion thresholds to be chosen based on the characteristics of each particular study (type of participants, design, resource constraints, etc.). In adult community samples, we recommend excluding participants with incomplete blocks or with more than 25% errors in ANTI trials, according to Luna et al. (2021a). Once the data have been processed, the main analysis consists of obtaining the score of the different indexes of the task for each participant.

**Figure 3:**
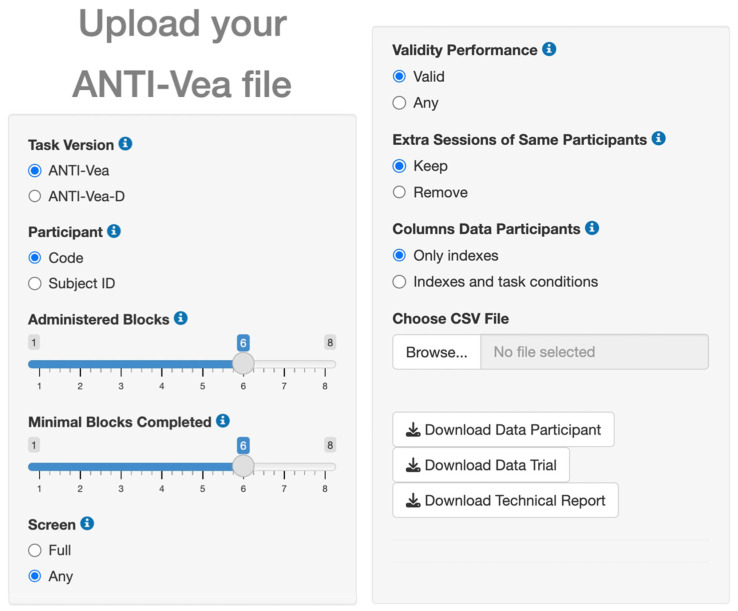
Shiny app to analyze the ANTI-Vea raw data (image adapted from the ANTI-Vea website, Analysis section). For the application to work correctly, it is necessary to set the parameters before uploading the file. Note that the current version of the app does not support ANTI-Vea versions with thought probes.

To support and facilitate the ANTI-Vea analysis process in obtaining the core indexes described in Table 1, we developed a code in R that is implemented in a Shiny app embedded in the Analysis section of the website (Figure 3). This app easily allows the transformation of a raw dataset of the ANTI-Vea into two clean and processed datasets: Data Participant and Data Trial, both in CSV format. In Data Participant, each row contains the information of a task session, with the columns including general information about the session (date of the session, noise, difficulty, trials and blocks completed, validity of the performance, etc.) as well as the scores of the ANTI-Vea core indexes in that session. Data Trial has the same structure as the raw dataset (i.e., trial-level rows) with additional columns related to the session. To do so, the user does not need any programming knowledge, but only click on the desired options for the following parameters: Task Version, Participant (column used to identify each participant), Administered Blocks per session, Minimal Blocks Completed (sessions with fewer completed blocks are removed), Screen (remove [Full] or retain [Any] sessions in which the screen was minimized by the participant), Validity Performance (remove [Valid] or retain [Any] sessions due to poor performance), Extra Sessions of the Same Participant, and Columns shown in the Data Participants file related to task indexes. The website includes sample CSV files for Data Participant and Data Trial, as well as their corresponding codebooks to ensure they are correctly interpreted.

Finally, the Shiny app includes the option to download a technical report (PDF format) of the whole analysis procedure and summary statistics of the task indices (see the website for a sample report).

For those with some programming skills, the R code underlying the Shiny app (default setting) is openly available in the “Analysis” section of the website. This format can be useful for a better understanding of the code and to facilitate modifications in the analysis flow (e.g., different filters and new indexes). Indeed, beyond the ANTI-Vea core indexes, there are several outcomes of the task that are worth considering. In this sense, the conditions that are manipulated to obtain the effects of the three attentional networks and the slope of decrement in vigilance can be specifically analyzed for a more detailed analysis (e.g., comparing congruent and incongruent conditions between two groups via a 2 × 2 mixed ANOVA). Having the conditions separated also allows us to check whether the task manipulation worked correctly, although this can also be checked by a one-sample *t*-test on the difference scores or slopes from the ANTI-Vea core indexes. Secondly, examples of new indexes that have been or may be derived from the core indices are the slope of cognitive control (Luna et al. 2022), mean and variability of RT in EV trials (Sanchis et al. 2020), scores from the Signal Detection Theory (SDT; i.e., sensitivity and response criterion; Luna et al. 2018), sequential effects such as post-error slowing and Gratton effect (Román-Caballero et al. 2021), scores from the psychometric-curve analysis (i.e., scale, shift, and lapse rate; Román-Caballero et al. 2023), between-blocks variability of vigilance scores, and scores from the diffusion decision model (i.e., drift rate, boundary separation, starting point, and non-decision component). We are in the process of implementing these extra scores into the R code. Suggestions for new additions to the code are welcome.

## 5. Discussion: Summary of Published Research with ANTI-Vea

The aim of the present tutorial was to provide a detailed, step-by-step user guide of the ANTI-Vea-UGR online platform (https://anti-vea.ugr.es/index.php), enabling researchers worldwide to freely collect, download, and analyze data using the ANTI-Vea task (Luna et al. 2018) and its adapted versions.

The ANTI-Vea is the latest version of the attentional networks test for measuring the functioning and interactions of the three attentional networks described by Posner and Petersen (1990). It combines different paradigms to assess phasic alertness, orienting, and executive control together. As the ANT task, it employs the typical flanker paradigm (Eriksen and Eriksen 1974) along with the spatial cueing task (Posner 1980) and the auditory tone used in the ANTI (Callejas et al. 2004). Thus, executive control is measured similarly with the ANT and the ANTI-Vea, although only congruent and incongruent trails are used (no neutral congruency trials are used in the ANTI-Vea, like in the ANTI). Attentional orienting is also measured with visual cues, like in the ANT. However, whereas a central vs. a 100% valid peripheral cue is used to measure orienting with the ANT, thus involving an important voluntary attention component, a peripheral non predictive cue is used to measure orienting with the ANTI-Vea, therefore perhaps indexing a more exogenous attention component. Finally, like in the ANTI version, an auditory warning signal is used to measure phasic alertness with the ANTI-Vea, instead of the visual cue used in the ANT.

Moreover, one of the most novel and important contributions of the ANTI-Vea is the theoretical distinction between two components of vigilance: EV, which refers to the ability to monitor and detect critical signals that rarely occur over a long period of time, and AV, understood as the capacity to maintain a fast response to any stimulus in the environment (Luna et al. 2018). Both components had already been described and tested separately with the MCT (Mackworth 1948) and the SART (Robertson et al. 1997) for EV, and with the PVT (Dinges and Powell 1985) for AV. However, the ANTI-Vea also succeeds in assessing the two vigilance components together in a single session.

When we analyze the vigilance decrement, this manifests as an increase in the mean and variability of response time for trials assessing AV where participants have to stop a millisecond counter as quickly as possible. In contrast, in EV trials, for which participants have to focus on and discriminate the vertical displacement of the central flanker (target), i.e., detect infrequent stimuli, the results show that there is no loss of sensitivity to these infrequent stimuli. What happens rather is that the participant’s response bias increases, according to what Thomson et al. (2016) state in their review. The interpretation that has been given to this phenomenon can be debated if we found a floor effect in FAs, an effect that is frequently observed in simple signal detection tasks such as the SART (Luna et al. 2021a). Nevertheless, to avoid this floor effect in the ANTI-Vea, which is a more complex task, FAs are only computed in those ANTI trials in which a FA response is more likely to be observed (Luna et al. 2021b).

A number of studies have been carried out since the implementation of this task (Cásedas et al. 2022; Coll-Martín et al. 2021; Feltmate et al. 2020; Hemmerich et al. 2023; Román-Caballero et al. 2021). Furthermore, the ANTI-Vea itself, or some studies that have used this task, have been featured in different dissemination reports. You can find more in the “Blog” section on the website.

When the participants perform this task online, on their own, there may be some potential difficulties that may cast doubt on the validity of the obtained data. Lighting conditions, distance to the screen, environmental noise, and the device features (operating system, screen size, etc.) may vary between participants. In addition, the participants may not understand the instructions and may not perform the task properly. Nevertheless, it is worth mentioning that vigilance has been successfully assessed in some other online studies, in which experimental conditions were not controlled as in typical studies in the lab (Claypoole et al. 2018; Fortenbaugh et al. 2015; Sadeh et al. 2011). Indeed, Luna et al. (2021a) concluded that the online ANTI-Vea was as effective as the standard ANTI-Vea carried out in the laboratory in assessing the functioning and interactions of the classical attentional components, along with EV and AV decrements. If you would like to monitor the conditions under which participants do the task, even if it takes a bit longer, you can make a video call to explain previously all the necessary conditions under which they have to perform the task or keep the video call while the participants carry out the task online from their house or other place meeting the desired experimental conditions (to ensure they do it correctly). Several studies (e.g., Cásedas et al. 2022) have also used the online ANTI-Vea, allowing them to reach large samples of participants from remote places and countries. In short, the use of this platform allows the research teams to investigate human attention in a simpler, cheaper, and more accessible way.

Thanks to the versatility offered by our online platform, the task and the different sub-versions can be applied to explain the variations and the functioning of attention in different populations, such as attention-deficit/hyperactivity disorder patients (e.g., Coll-Martín et al. 2021, 2023), athletes (Huertas et al. 2019), and musicians (Román-Caballero et al. 2021), and to apply it under multiple conditions (e.g., caffeine intake and exercise intensity; Sanchis et al. 2020) or states (e.g., fatigued, relaxed, mindful, excited; see, for instance, Feltmate et al. 2020).

In summary, the online ANTI-Vea task can be run with standard parameters or adapted to the specific needs of different research questions. It is in continuous development to adapt new versions and functionalities to the task, such as the measurement of distractions or monitoring whether the participants are focused on the task or engaged in mind-wandering.

## 6. Conclusions

In conclusion, the ANTI-Vea-UGR platform provides a rigorous, accessible, and free assessment of the attentional functioning, encompassing the three attentional networks and two vigilance components. These functions, grounded in influential theoretical frameworks and extensive empirical research, are measured with a reasonable reliability in the ANTI-Vea, the main task of the platform. The resources for online data collection adapted to different languages and analysis through a user-friendly app facilitate task administration by different researchers and in diverse contexts and populations. Finally, the platform’s free nature aligns with open science principles, while being supported by a public institution that ensures proper data protection. Therefore, we encourage researchers to take advantage of this valuable resource to advance the study of attention across different areas.

## Figures and Tables

**Table 1:** ANTI-Vea core indexes. Adapted from https://anti-vea.ugr.es/analysis.html.

Domain	Index	Description	Observed Results (In-Lab/Online Version) *M* (*SD*) *
Attentional networks (ANTI)	Overall RT	Mean correct RT across all ANTI trials.	629 ms (98)/652 ms (98)
	Overall errors	Percentage of errors across all ANTI trials.	6.10% (4.74)/5.95% (4.36)
	Alerting RT	RT difference between *No Tone* and *Tone* conditions in trials with no cue.	40 ms (26)/37 ms (43)
	Alerting errors	Error difference between *No Tone* and *Tone* conditions in trials with no cue.	2.42% (4.79)/1.46% (4.75)
	Orienting RT	RT difference between *Invalid* and *Valid* conditions.	40 ms (27)/46 ms (27)
	Orienting errors	Error difference between *Invalid* and *Valid* conditions.	−0.07% (3.76)/0.44% (3.98)
	Congruency RT	RT difference between *Incongruent* and *Congruent* conditions.	43 ms (27)/41 ms (33)
	Congruency errors	Error difference between *Incongruent* and *Congruent* conditions.	0.81% (4.70)/0.36% (3.88)
Executive vigilance (EV)	Hits	Percentage of times the displacement of the central arrow is correctly detected by pressing the spacebar. Synonymous with 1 minus omission errors or misses.	73.24% (17.34)/78.87% (14.04)
	Hits slope	Linear slope of hits over blocks, which tends to decrease.	−1.89% (3.64)/−1.93% (3.61)
	False alarms	Percentage of times the spacebar is pressed when there is no substantial displacement of the central arrow. Synonymous with commission errors.	6.35% (5.80)/6.88% (6.02)
	False alarms slope	Linear slope of false alarms over blocks, which tends to decrease.	−0.27% (0.94)/−0.23% (1.23)
Arousal vigilance (AV)	Mean RT	Average time to stop the red down counter.	491 ms (62)/509 ms (85)
	Mean RT slope	Linear slope of mean RT over blocks, which tends to increase.	4 ms (11)/5 ms (14)
	*SD* RT	Response speed variability to stop the red down counter.	90 ms (39)/83 ms (32)
	*SD* RT slope	Linear slope of *SD* RT over blocks, which tends to increase.	4 ms (11)/6 ms (13)
	Lapses	Percentage of times with an excessively large (RT > 600 ms) or no response to the red down counter.	11.35% (14.57)/13.19% (17.53)
	Lapses slope	Linear slope of lapses over blocks, which tends to increase.	1.47% (3.32)/1.67% (3.73)

* The content in this column represents the weighted average of the results in Cásedas et al. (2022; online version), Coll-Martín et al. (2021; in-lab version), and Luna et al. (2021a; both in-lab and online versions), with an overall sample of 427 participants for the in-lab version and 522 participants for the online version. The samples of these studies consisted of university students.

**Table 2:** Internal consistency scores of the ANTI-Vea indexes across studies and task versions.

Task Index	In-Lab Reliability (*r*_SB_)		Online Reliability (*r*_SB_)	
	Luna et al. (2021a)	Coll-Martín et al. (2021)	Luna et al. (2021a)	Cásedas et al. (2022)
*N*	314	113	303	219
Attentional networks				
Overall RT	.99	.99	.99	.99
Overall errors	.92	.91	.89	.91
Alerting RT	.22	.47	.36	.45
Alerting errors	.18	.51	.11	.24
Orienting RT	.31	.36	.30	.40
Orienting errors	.60	.26	.28	.22
Congruency RT	.67	.66	.68	.64
Congruency errors	.66	.60	.52	.51
Executive vigilance				
Hits	.94	.94	.92	.91
Hits slope		.27		.58
False alarms	.85	.85	.79	.78
False alarms slope		.40		.21
Arousal vigilance				
Mean RT	.98	.97	.99	.96
Mean RT slope		.75		.65
*SD* RT	.84	.88	.76	.71
*SD* RT slope		.54		.65
Lapses	.96	.96	.98	.96
Lapses slope		.78		.81

*Note*. *r*_SB_ = Spearman–Brown split-half reliability coefficient.

**Table 3:** Setting parameters and all the possible values to create your own link of the task to share with your participants.

Setting Parameter (Parameter = Default Value)	Description and Setting Values
lang = en	Language of instructions: “de” for German, “en” for English, “es” for Spanish, “fr” for French, “it” for Italian, and “pl” for Polish.
type = ANTI_VEA	Specific task to be performed: ANTI_VEA, ANTI, SART, PVT, SART-PVT, ANTI-Only, SART-Only-Go, SART-Only-NoGo, PVT-Only, ANTI-Vea-D.
pc = 1234	Participant code; only numbers allowed here. Any combination of digits is fine. If this parameter is not specified, the task does not start.
exp = Power_ANTI-Vea	The name of your experiment.
gr = Exp	The name of the experimental group, in case there is one.
no = 2	Noise: this parameter refers to the random variability of the spatial position of the arrows (1–6); the default value is 2, keep it if you are not interested in this manipulation.
dif = 2	Difficulty: this parameter manipulates the perceptual salience of the target and therefore affects EV. It refers to the spatial distance of the central arrow in relation to the adjacent arrows; 1 (most difficult) to 5 (less difficult) values are allowed; the default value is 2, keep it if you are not interested in this manipulation.
st = 200	Target display duration: integers from 0 to 1700 ms are accepted values, 200 ms being the value in the standard version of the task.
dP = false	This value should be set to “true” if you want participants to do the whole practice blocks before the experimental blocks, and to “false” if you want them to go straight to the experimental blocks, with just a reminder of the instructions. This feature is useful when collecting data from several sessions in within-subjects designs.
B = 6	Number of experimental blocks (1–8); the value can be set to 0 if you want the participants to only run the practice, with no experimental block; 6 is the number of blocks by default.
probes = 0	This parameter refers to the number of thought probes (TP) used to measure mind wandering. Depending on the value given to this variable TPs are presented 4, 8, or 12 times per block. By default, the standard version of the task does not include any thought probes. Leave this parameter at 0 to run the standard version of the task, without thought probes.

Link example: https://anti-vea.ugr.es/Sitio_web/ANTI-Vea1/Anti.html?lang=en&type=ANTI_VEA&pc=1234&exp=Power_ANTI-Vea&gr=Exp&no=2&dif=2&st=200&dP=true&B=6&probes=0.

## Data Availability

No new data were collected during the preparation of this article.

## Notes

1 Technically, a decrement in sensitivity across the blocks of the ANTI-Vea has been also observed. However, compared to the change in response bias, that effect is substantially lower and likely due to a floor effect in FAs (Luna et al. 2021a) or other artifacts (Román-Caballero et al. 2023).

2 In order for the system to assign the same Subject ID to two or more different sessions, the codes for Participant, Name, Experiment, and Group must match (in older versions, the access cookies also need to match). Therefore, in studies where participants perform multiple sessions of the task, it is recommended that the experimenter distinguish their participants by using the Participant Code instead of the Subject ID.
